# Conjugated Bilirubin Differentially Regulates CD4+ T Effector Cells and T Regulatory Cell Function through Outside-In and Inside-Out Mechanisms: The Effects of HAV Cell Surface Receptor and Intracellular Signaling

**DOI:** 10.1155/2016/1759027

**Published:** 2016-08-04

**Authors:** Karla F. Corral-Jara, Jorge L. Trujillo-Ochoa, Mauricio Realpe, Arturo Panduro, Juan F. Gómez-Leyva, Yvonne Rosenstein, Alexis Jose-Abrego, Sonia Roman, Nora A. Fierro

**Affiliations:** ^1^Unidad de Inmunovirología, Servicio de Biología Molecular en Medicina, Hospital Civil de Guadalajara “Fray Antonio Alcalde”, 44280 Guadalajara, JAL, Mexico; ^2^Departamento de Biología Molecular, Centro Universitario de Ciencias de la Salud, Universidad de Guadalajara, 44100 Guadalajara, JAL, Mexico; ^3^Departamento de Fisiología, Centro Universitario de Ciencias de la Salud, Universidad de Guadalajara, 44100 Guadalajara, JAL, Mexico; ^4^Departamento de Medicina Veterinaria, Centro Universitario de Ciencias Biológicas y Agropecuarias, Universidad de Guadalajara, 44100 Guadalajara, JAL, Mexico; ^5^Servicio de Biología Molecular en Medicina, Hospital Civil of Guadalajara “Fray Antonio Alcalde”, 44280 Guadalajara, JAL, Mexico; ^6^Departamento de Clínicas Médicas, Centro Universitario de Ciencias de la Salud, Universidad de Guadalajara, 44100 Guadalajara, JAL, Mexico; ^7^Instituto Tecnológico de Tlajomulco, 45640 Tlajomulco, JAL, Mexico; ^8^Departamento de Medicina Molecular y Bioprocesos, Instituto de Biotecnología, Universidad Nacional Autónoma de México, 62210 Ciudad de México, Mexico

## Abstract

We recently reported an immune-modulatory role of conjugated bilirubin (CB) in hepatitis A virus (HAV) infection. During this infection the immune response relies on CD4+ T lymphocytes (TLs) and it may be affected by the interaction of HAV with its cellular receptor (HAVCR1/TIM-1) on T cell surface. How CB might affect T cell function during HAV infection remains to be elucidated. Herein,* in vitro* stimulation of CD4+ TLs from healthy donors with CB resulted in a decrease in the degree of intracellular tyrosine phosphorylation and an increase in the activity of T regulatory cells (Tregs) expressing HAVCR1/TIM-1. A comparison between CD4+ TLs from healthy donors and HAV-infected patients revealed changes in the TCR signaling pathway relative to changes in CB levels. The proportion of CD4+CD25+ TLs increased in patients with low CB serum levels and an increase in the percentage of Tregs expressing HAVCR1/TIM-1 was found in HAV-infected patients relative to controls. A low frequency of 157insMTTTVP insertion in the viral receptor gene* HAVCR1/TIM-1* was found in patients and controls. Our data revealed that, during HAV infection, CB differentially regulates CD4+ TLs and Tregs functions by modulating intracellular pathways and by inducing changes in the proportion of Tregs expressing HAVCR1/TIM-1.

## 1. Introduction

Bilirubin (BR), long considered to be exclusively a toxic waste product, has recently been recognized as an immune-modulatory metabolite able to modulate CD4+ T lymphocyte (TL) function [1–3]. Particularly, we recently reported an immune-modulatory role of conjugated BR (CB) in hepatitis A virus (HAV) infection [1]. BR can suppress inflammation and increase antioxidant enzyme generation in activated neonatal neutrophils by downregulating the lipopolysaccharide- (LPS-) induced generation of IL-8 [4]. Moreover,* in vitro* models reveal that BR concentrations >25 *μ*M modulate CD4+ T cell and neutrophil apoptosis [3, 4]. The induction of tolerance reported after the administration of BR to transplant recipients, which results from the* de novo* generation of T regulatory cells (Tregs) in murine models [5, 6], is in agreement with the ability of BR to inhibit T cell proliferation and to decrease IL-2 production in human lymphocytes [1]. Furthermore, a BR-conferred protection against autoimmune diseases has been described [3, 7, 8], presumably as a result of the capacity of BR to bind to the peptide binding groove of human leukocyte antigen (HLA) molecules, blocking the antigenic peptide presentation to T cell receptor (TCR) and hence suppressing autoimmune responses [9]. This is consistent with the fact that BR treatment results in downregulation of inducible MHC class II expression, affecting Ag presentation to CD4+ T cells [3], and supports that, under pathological circumstances, changes in normal BR concentration may modulate specific immune responses through specific receptors.

Infections with hepatotropic viruses cause an elevation of serum aminotransferase activity and of serum-associated BR [1, 10, 11]. Viral hepatitis A is a major health concern worldwide, with a higher incidence in developing countries. Although improved hygiene and vaccination have reduced the HAV infection rate, the virus remains widespread and the infection is generally acquired in early childhood [12]. HAV is also considered a foodborne pathogen, based on the documented outbreaks of infection caused by the consumption of frozen fruits in developed countries [13–15], as well as a major cause of acute liver failure and transplant in pediatric patients [16]. The progression of HAV infection is restricted by the host immune response [10], which may also be affected by host-metabolic components. Particularly, during the final stages of HAV infection, heme degradation is interrupted, leading to the deregulation of BR internalization and excretion by hepatocytes, which results in increased CB values (0.3 to 6 mg/dL) [11].

We recently reported that CB plays a role in adjusting STAT-1 and STAT-5 function and in determining cytokine profiles during HAV infection [17, 18]. The fact that a TGF-beta-associated anti-inflammatory cytokine profile is observed in HAV-seropositive pediatric patients with low serum values of CB (<2 mg/dL) suggests a potential role for Tregs in the clinical courses induced by HAV. Conversely, a proinflammatory profile is found in patients with higher serum CB levels (>2 mg/dL) [18, 19]. This is consistent with the temporary inhibition of Tregs function described during infection, which has been explained in terms of a specific interaction between HAV and its cellular receptor HAVCR-1/TIM-1 on the T cell surface [20]. Interestingly, a six-amino-acid insertion in the* HAVCR1/TIM-1* (157insMTTTVP) gene is associated with the development of severe HAV infection [21]. Although data from several reports indicate that this receptor, together with TCR and costimulatory signals, regulate the expansion and effector functions of T helper cells [22], the functions and mechanisms through which HAVCR1/TIM-1 may be regulating T cell activity are poorly understood. Particularly, whether specific metabolic products, such as BR, might affect these mechanisms during the course of HAV infection remains to be elucidated.

## 2. Materials and Methods 

### 2.1. Reagents

CB was from Merck-Millipore, Darmstadt, Germany. Anti-CD3 mAb, anti-CD28 mAb, anti-phosphotyrosine (anti-pTyr) PY20-Alexa 488, anti-CD4-Alexa 488, anti-CD25-PercP, anti-FOXP3-Alexa 488, and anti-TIM-1-PE were from Biolegend, San Diego, CA, United States. Rabbit anti-mouse-IgG1 and IgG2a were from Fisher Biotec, Hampton, New Hampshire, United States. Carboxyfluorescein diacetate succimidylester (CFSE) was from Biolegend. The 7-Plex T-Cell Receptor Th17 and IL-17 Magnetic beads MAGPIX Kits were from Merck-Millipore. Treg (CD4+CD25+CD127^low^) and CD4+ T cell isolation kit were from Miltenyi Biotec, Bergisch Gladbach, Germany. The primers were from IDT, Coralville, Iowa, United States. The DNA Extraction and Purification Kit and Gel and PCR Clean-up System were from Promega, Madison, Wisconsin, United States. Recombinant Taq DNA Polymerase was from Thermo Fisher Scientific, Waltham, Massachusetts, United States, and QIA Quick PCR purification kit was from Qiagen, Hilden, Germany.

### 2.2. *In Vitro* Analysis

#### 2.2.1. Cell Purification

Ficoll-Paque PLUS (Healthcare, Uppsala, Sweden) gradient centrifugation was used to isolate peripheral blood lymphoid cells (PBLCs) from anticoagulated blood samples of pediatric healthy donors as described previously [17]. The buffy coat of each sample was washed three times with PBS (300 ×g; 10 min; room temperature) and resuspended in autoMACS Running Buffer (Miltenyi Biotec). CD4+ TLs were purified by negative magnetic selection with the CD8, CD19, CD123, and CD127 antibodies followed by Treg (CD4+CD25+) positive selection with anti-CD25 Micro Beads (Miltenyi Biotec). Before experimentation, CD4+ TLs and Tregs were arrested for 2 h in RPMI 1640 (HyClone, Logan, UT) supplemented with 2% (v/v) fetal calf serum (FCS) with 2 mM L-glutamine, 50 *μ*g/mL penicillin, 50 *μ*g/mL streptomycin, and 50 *μ*M *β*-mercaptoethanol (Sigma, St Louis, MO).

#### 2.2.2. Phosphotyrosine Intracellular Staining

Purified CD4+ TLs (5 × 10^6^) from pediatric healthy donors were incubated according to different protocols. Cells were stimulated with anti-CD3 (1 *μ*g/mL) and anti-CD28 (1 *μ*g/mL) antibodies (Biolegend) with or without various doses of CB (0.3, 2 or 15 mg/dL) in 1 mL RPMI 1640 medium supplemented with 10% FCS and 5% CO_2_ at 37°C for 30 minutes. Nonstimulated cells were included as a negative control. After the incubation, the cell pellet was obtained by centrifugation (300 ×g; 10 min; room temperature) and resuspended in 100 *μ*L of 1x fixation/permeabilization buffer (Biolegend Fix/Perm Buffer). Cell permeabilization was performed for 30 min at 4°C under dark conditions. The cells were then washed twice by centrifugation and permeabilized by adding 50 *μ*L of 1x permeabilization buffer (Biolegend Perm Buffer). Then, cells (1 × 10^6^) were resuspended in 100 *μ*L of assay buffer (Biolegend) and incubated with 2.0 *μ*L of anti-phosphotyrosine PY20-Alexa 488 antibody (30 min; 4°C) while being protected from light. The cells were then washed twice by centrifugation (300 ×g; 10 min), resuspended in 250 *μ*L PBS, and analyzed using a GUAVA EASYCYTE 6 with INCYTE 2.0 software (Merck-Millipore). The median fluorescence intensity (MFI) was obtained from the acquisition of 10,000 events of triplicate counts of 1 × 10^6^ cells.

#### 2.2.3. Treg Cell Suppression Assay

A total of 1 × 10^6^ Tregs (CD4+CD25+FOXP3+) per condition purified from pediatric healthy donors were preincubated with increasing concentrations of CB (0, 0.3 and 2 mg/dL), in a final volume of 300 *μ*L of supplemented RPMI (5% fetal calf serum (FCS) + 5% bovine calf serum (BCS)) for 72 h at 5% CO_2_. Following which, Tregs were recovered and cocultured with CD4+CD25− effector T cells (Teff) previously labeled with 2 *μ*M CFSE at 37°C for 10 min. Cocultures of 1 : 2 ratio of Teff : Tregs (preincubated with or without CB), at 37°C and 5% CO_2_, were performed in final volume of 300 *μ*L/well, in RPMI 1640 supplemented with 5% fetal calf serum (FCS) + 5% bovine calf serum (BCS) and stimulated with anti-CD3 (1 *μ*g/mL) and anti-CD28 (1 *μ*g/mL). After 7 days of coculture, the cells were harvested and cell proliferation was evaluated by flow cytometry. The proliferation of Teff stimulated with anti-CD3 (1 *μ*g/mL) and anti-CD28 (1 *μ*g/mL) antibodies in the absence of Tregs was used as a positive control. The percent of suppression was determined with the following formula: (mean final Teff cell number − mean final Teff cell number incubated with Treg cells)/(mean final Teff cells number) × 100.

#### 2.2.4. Percentage of Tregs Expressing TIM-1

A total of 400,000 Tregs per condition from pediatric healthy donors were stimulated with anti-CD3 (1 *μ*g/mL), anti-CD28 (1 *μ*g/mL), and different doses of CB (0.3, 2, or 15 mg/dL) in a total volume of 1 mL of RPMI 1640 (Hyclone) (10% FCS) for 72 h at 37°C and 5% CO_2_. Nonstimulated cells were included as a negative control. Cells were recovered and stained with 2.0 *μ*L of TIM-1-PE antibody for 20 min at 2–8°C under dark conditions. Finally, the percentage of Tregs TIM-1+ cells was determined by flow cytometry. Three independent experiments were performed.

#### 2.2.5. IL-17 Cytokine Profile

Purified CD4+ TLs (1 × 10^6^) from pediatric healthy donors were incubated according to different protocols. Cells were stimulated with anti-CD3 (1 *μ*g/mL) and anti-CD28 (1 *μ*g/mL) antibodies in absence of presence of various doses of CB (0.3, 2 or 15 mg/dL) in 1 mL RPMI 1640 medium supplemented with 10% FCS and 5% CO_2_ at 37°C for 72 hours. Nonstimulated cells were included as a negative control. After the incubation, cell supernatant (100 *μ*L) was recovered and clarified by high-speed centrifugation. We used a 1-plex kit (IL-17) from Merck-Millipore, and cytokine analysis was performed using a MAGPIX system powered by xMAP Luminex Technology with the xPONENT® software of EMD (Merck-Millipore). The assay was performed according to the supplier's instructions. Briefly, following the prewetting of each plate, 50 precombined beads of IL-17 were added and washed twice. Cell supernatants (25 *μ*L) from distinct stimulation conditions were diluted 1 : 2 with the assay buffer and added to the plate. The plate was shaken for 30 s at 300 g and then incubated overnight on a plate shaker at 300 g at room temperature. The plate was washed twice, 25 *μ*L of detection antibody was added per well, and the plate was then incubated for one hour on a plate shaker. Subsequently, 50 *μ*L of a streptavidin-PE conjugate was added per well and incubated for 30 min at room temperature. Finally, the plate was washed three times, 150 *μ*L of sheath fluid was added to each well, and the plate was read by MAGPIX machine (Merck-Millipore).

### 2.3. Ex Vivo Analysis 

#### 2.3.1. Study Population

A total of 315 unrelated subjects from South and West México were included in this study, 156 of which were pediatric patients (<15 years old) with acute HAV infection (85 sera samples from South México pediatric patients recruited during 2011 and 71 blood samples from West México pediatric patients recruited during May 2015 to February 2016). The remaining subjects included 60 pediatric healthy controls and 99 healthy adult donors (18–50 years old) from West México. The study was conducted at the Centro de Referencia de Hepatitis Virales del Occidente de México and the Unidad de Inmunovirologia in the Servicio de Biologia Molecular, Hospital Civil de Guadalajara, Fray Antonio Alcalde (HCFAA), in Guadalajara, Jalisco, México.

Patients and healthy donors with liver disease who were undergoing treatment with a hepatotoxic drug, those with acute or chronic hepatitis E virus (HEV), hepatitis B virus (HBV), or hepatitis C virus (HCV) infections, and those diagnosed with autoimmune hepatitis were excluded from the study. None of the pediatric patients and pediatric healthy controls included in the study had been vaccinated against HAV and HBV. After the healthy donors and children's parents had provided informed consent, blood samples were obtained by venipuncture. The Ethical Committees of the HCFAA and the Centro Universitario de Ciencias de la Salud, Universidad de Guadalajara, approved this study.

#### 2.3.2. Clinical and Demographic Data

Hepatitis was defined as hepatomegaly, fever (>38°), and/or jaundice with elevated values of serum AST (>38 IU/L) and ALT (>35 IU/L), as previously described [18]. Additionally, CB (>0.3 mg/dL) and albumin values were measured and demographic and clinical features were recorded using a structured questionnaire, as previously reported [18].

#### 2.3.3. Serological Tests

To detect acute hepatitis A infection, serum samples from pediatric patients diagnosed with hepatitis were screened for the presence of anti-HAV IgM and the absence of anti-HAV IgG. All samples were negative for antibodies to HBV, HCV, and HEV. The presence of anti-HAV IgM and the absence of anti-HAV IgG, the surface antigen of HBV (HBsAg), and anti-HCV antibodies were tested by using a third-generation microparticle immunoenzymatic assay (AxSYM HAVAB-M 2_0, AxSYM HBsAg (V2), and AxSYM HCV 3.0; Abbott Laboratories, Chicago, IL) with an AxSYM analyzer (Abbott Laboratories). Total anti-hepatitis B core antigen anti-HBc (total IgM and IgG) and anti-HEV antibodies were measured by using immunoenzymatic assays (Monolisa Anti-HBc PLUS, Bio-Rad Laboratories, Chicago, IL, MP Diagnostics, Geneva, Switzerland and MyBiosource, San Diego, CA, USA, resp.) with a PR 3100 TSC analyzer (Bio-Rad). The levels of albumin/globulin, ALT, AST, alkaline phosphatase, total protein, total BR, and CB were measured in the serum samples, following routine clinical laboratory procedures.

#### 2.3.4. Liver Injury Categorization in HAV-Infected Children

Pediatric patients who tested positive for acute HAV infection (anti-HAV IgM^+^ and anti-HAV IgG^−^) and negative for antibodies to HBV, HCV, and HEV and who exhibited abnormal levels of ALT and AST (>38 IU/L and/or >35 IU/L, resp.) were categorized as follows and detailed in Table 1:

 Patients who exhibited CB levels > 0.3 mg/dL to 2 mg/dL.
 Patients who exhibited CB levels > 2 mg/dL.

*Healthy Controls (H)*. Children with normal hepatic enzymatic activity in the absence of HAV, HEV, HBV, and HCV serological markers.

#### 2.3.5. TCR Signaling Pathway

The cell lysates of purified CD4+ T cells (5 × 10^6^) from 16 healthy pediatric donors, 11 pediatric patients infected with HAV with serum CB levels between 0.3 and <2 mg/dL, and 7 pediatric patients infected with HAV with serum CB levels > 2 mg/dL were analyzed to detect TCR pathway proteins phosphorylation. Cells were lysed with 100 *μ*L of lysis buffer (100 mM Hepes of pH 7.5, 1 M MgCl_2_, 3 M NaCl, 1 mM EDTA, Triton X-100 and a protease inhibitor cocktail of PMSF, BGP, NAF, NaVO_4_, leupeptin, antipain, aprotinin, and DTT). Then, cell lysates were incubated for 30 min at 4°C with stirring, and finally, supernatant was obtained by centrifugation (300 ×g; 10 min; 4°C). The protein supernatant content was estimated by a microwell plate version of the Bradford method. The cell lysates were analyzed with a 7-plex T cell receptor kit (CD3 epsilon, CREB, ERK MAP 1/2, LAT, LCK, SYK, and ZAP-70 phosphoproteins) with a MAGPIX system powered by xMAP Luminex Technology with the xPONENT® software. Briefly, following the prewetting of each plate for 10 min with assay buffer, 25 *μ*L of cell lysates (20 *μ*g of protein) and 25 *μ*L of precombined beads of all the 7 individual proteins were added to the plates. The plates were shaken and incubated overnight at 4°C while protected from light. Then, the plates were washed twice, 25 *μ*L of detection antibody was added to each well, and the plates were further incubated for one hour on a plate shaker while protected from light. Detection antibody was removed and subsequently, 50 *μ*L of a streptavidin-PE conjugate was added per well and incubated for 15 min at room temperature while protected from light. A total of 25 *μ*L of Amplification buffer was added per well, and plates were incubated 15 min at room temperature while protected from light. Finally, the plate was washed three times to remove streptavidin-PE and Amplification buffer and, beads in each well were resuspended with 150 *μ*L of assay buffer. The plate was read by MAGPIX machine. HeLa and stimulated Jurkat cell lysates were used as negative and positive controls, respectively. At least 50 events per bead were read for each sample in triplicate wells.

#### 2.3.6. Th17 Cytokine Profile Analysis

Before cytokine evaluation, sera from pediatric healthy donors and HAV-seropositive patients were first clarified by high-speed centrifugation. We used a 5-plex kit (IL-6, IL-21, IL-22, and macrophage inflammatory protein 3*α* (MIP-3*α* (CCL20)) and IL-17F) from Merck-Millipore, and multicytokine analysis was performed using a MAGPIX system. The assays were performed as described before.

#### 2.3.7. CD4-CD25 Staining

PBLCs of 32 pediatric patients coursing the acute phase of HAV infection and 17 pediatric healthy donors were costained with anti-CD25+ and anti-CD4+ antibodies. Briefly, 1 × 10^6^ cells were resuspended in 100 *μ*L of assay buffer (Merck-Millipore) and incubated with 2.0 *μ*L of anti-CD4-Alexa 488, anti-CD25-PercP antibodies (30 min; 2–8°C) while protected from light. The cells were then recovered by centrifugation (300 ×g; 5 min), resuspended in assay buffer, and evaluated by flow cytometry. The percentage of positive cells was obtained from the acquisition of 10,000 events. Triplicate counts from the 1 × 10^6^ cells resuspended in assay buffer were conducted.

#### 2.3.8. CD4-CD25-TIM-1 Staining

A total of 1 × 10^6^ PBLCs per condition from 17 HAV-seropositive pediatric patients and 25 pediatric healthy donors were incubated with 2.0 *μ*L of Alexa Fluor anti-CD4 antibody, 2.0 *μ*L of anti-CD25-PE, and 2.0 *μ*L of anti-PerCP TIM-1 in dark conditions at 2–8°C for 30 min. The cells were then recovered by centrifugation at 300 ×g for 5 min, resuspended in assay buffer, and analyzed by flow cytometry. The percentage of positive cells was obtained from the acquisition of 10,000 events. Triplicate counts from the 1 × 10^6^ cells resuspended in assay buffer were conducted.

#### 2.3.9. Evaluation of Polymorphism 157insMTTTVP on HAVCR1/TIM-1 Gene Locus

Total genomic DNA was purified from PBLC samples obtained from 99 unrelated donors and 21 HAV-infected pediatric patients. PCR and subsequent DNA sequencing of 294 bp covering exon 4 of gene encoding* HAVCR1/TIM-1* HAV receptor allowed detecting the 157insMTTTVP polymorphism as previously described [23]. Briefly, total genomic DNA was extracted using Wizard Genomic DNA Purification Kit following manufacturer instructions and used as template for PCR. Oligonucleotides used were Forward 5′-GGG CAA TGA CCA AGA TTG AC-3′ and Reverse 5′-ACC TTG ATA CAA TGC CCT GG-3′ [24]. PCR reactions were performed in a 50 *μ*L reaction volume, containing 20–50 ng DNA, 2 mM MgCl_2_, 1x reaction buffer (Promega, MA), 0.2 mM dNTPs (Invitrogen, CA), 0.4 mM of each oligonucleotide, and 0.4 U of Taq DNA Polymerase. PCR reactions were incubated in a Techne Endurance Tc-300 system (Staffordshire, UKA) by 35 cycles of 94°C/1 min, 55°C/1 min and 72°C/1 min. Amplified DNA products were separated in 1.5% agarose gels and visualized by ethidium bromide staining. DNA fragments of the expected length were extracted from gel using Wizard PCR Clean-Up system, quantified in a NanoDrop 2000 spectrophotometer (Thermo Scientific), and sequenced bidirectionally using BigDye Terminators v2.0 Cycle Sequencing Kit (Applied Biosystems ABI, Foster City, CA) following manufacturer instructions. Sequencing reactions were run on an automated DNA sequencer ABI PRISM 3700 (ABI). Electropherograms obtained from sequencing files were edited with the BioEdit software (http://www.mbio.ncsu.edu/bioedit/bioedit.html. Carlsbad, CA) and converted into fasta files. Sequences were assembled and compared with reference sequences retrieved from GenBank by using MEGA software version 6.0 (Molecular Biology and Evolution 30: 2725–2729). A multisequence alignment file was obtained as final dataset using ClustalW algorithm with default settings, and allowed detecting the presence of the 18-nt gene insertion based on comparison with the sequences downloaded from GenBank. The reference sequences from GenBank were AF043724 (wild-type: no insertion) and CR457114 as an example of the 18-nt gene insertion as reported [23].

### 2.4. Statistical Analysis

Data are presented as the mean or medians and standard deviation (SD). Statistical comparisons were performed by using Graph Pad Prism software version 5.01 (Graph Pad Software, Inc., San Diego, CA). Nonparametric Kruskal-Wallis and Mann-Whitney *U* tests for comparisons between groups were used to calculate the statistical significance of the assay results. A *P* value < 0.05 was considered statistically significant. Post hoc methods were used to ensure that there were differences between the compared groups. To study associations between variables, the Spearman correlation coefficients were calculated.

## 3. Results

### 3.1. CB Reduces the Degree of Tyrosine Phosphorylation in CD4+ TLs

**Figure 1:**
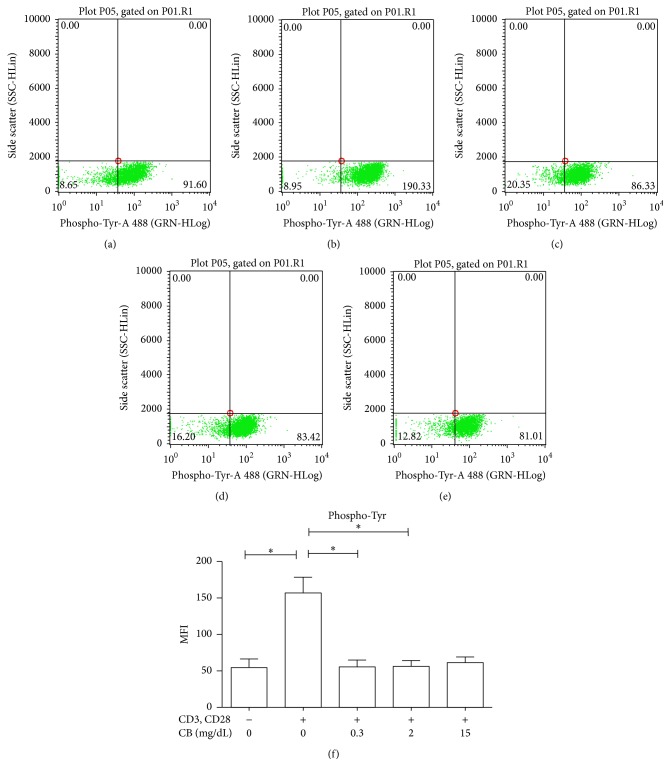
CB causes a decrease in the degree of tyrosine phosphorylation in human CD4+ TLs* in vitro*. Purified CD4+ TLs from pediatric healthy donors were incubated at 37°C, 5% CO_2_, 30 min, under different conditions: (a) TLs without stimulus. (b) TLs with anti-CD3 and anti-CD28. (c) TLs with anti-CD3, anti-CD28, and 0.3 mg/dL CB. (d) TLs with anti-CD3, anti-CD28, and 2 mg/dL CB. (e) TLs with anti-CD3, anti-CD28, and 15 mg/dL CB. Cells were subsequently recovered and stained with an anti-pTyr mAb and then analyzed by using flow cytometry. Representative dot plots are shown (a–e). (f) The medians and standard deviations of three repetitions are presented. Nonparametric Kruskal-Wallis for comparison between groups was used to calculate statistical significance. *P* < 0.05 was considered statically significant. ^*∗*^ *P* < 0.05.

CD4+ TLs were treated with CB, and the degree of tyrosine phosphorylation was assessed to determine the effect of this metabolite on the overall signaling capacity of the cells. As expected [25, 26], we observed an increased MFI of phosphorylated tyrosine in CD4+ TLs stimulated with anti-CD3 and anti-CD28 antibodies (157.2 ± 58.23) as compared with cells that were not stimulated (54.90 ± 32.03) (Figures 1(a) and 1(b)). However, treating the cells with clinically relevant concentrations of CB in conjunction with CD3 and CD28 engagement resulted in decreased intracellular tyrosine phosphorylation as compared to CD3- and CD28-stimulated cells (Figures 1(c)–1(f)). Together, these data suggest that CB causes changes in the intracellular signaling pathways of TLs.

### 3.2. CB Modifies the Degree of CD3-Epsilon, SYK, and CREB Phosphorylation in CD4+ TLs of HAV-Seropositive Pediatric Patients

**Figure 2:**
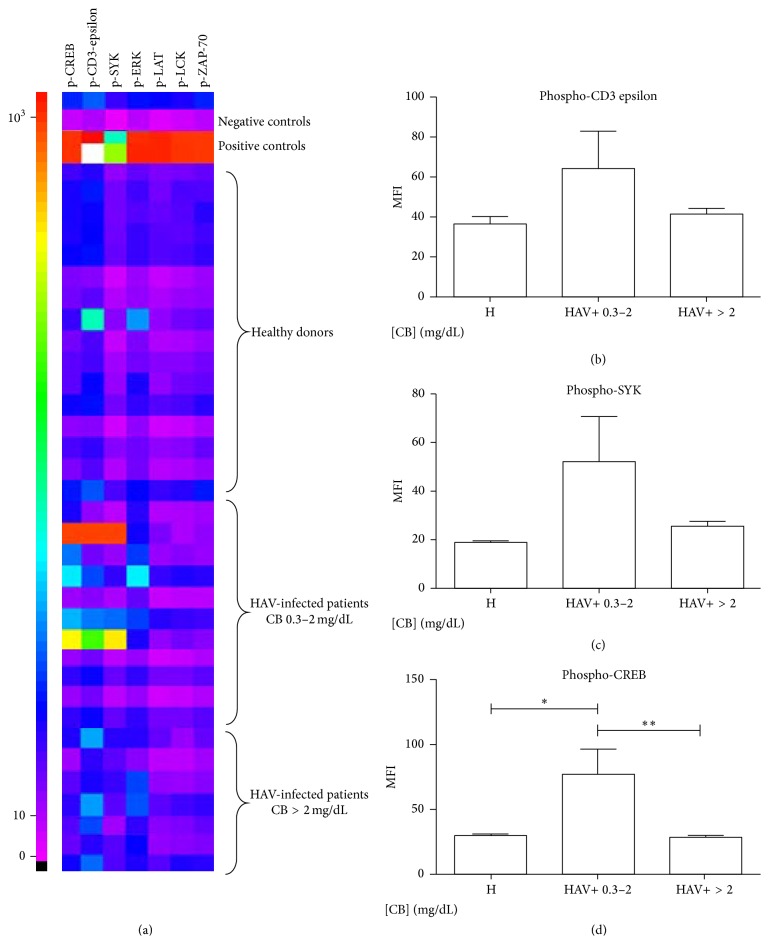
CD4+ TLs from HAV-seropositive patients with CB levels greater than 2 mg/dL have a lower degree of CD3 epsilon, CREB, and SYK phosphorylation. TCR-related intracellular signals were evaluated in CD4+ TLs lysates from pediatric patients and healthy donors by MAGPIX Technology. Representative Heat map (MFI) is shown in (a). The medians of the MFI and SD of phosphorylated CD3 epsilon (b), phosphorylated SYK (c), and phosphorylated CREB (d) from HAV+ patients with CB 0.3–2 mg/dL (*n* = 11), HAV+ patients with CB > 2 mg/dL (*n* = 7), and healthy (H) donors (*n* = 16) are shown. Nonparametric Kruskal-Wallis for comparison between groups was used to calculate statistical significance. *P* < 0.05 was considered statically significant. ^*∗*^ *P* < 0.05 and ^*∗∗*^ *P* < 0.001.

The TCR signaling pathway is an intracellular signaling pathway specifically related to the activity and function of T cells. The MFIs of phosphorylated CREB, CD3-epsilon, SYK, ERK, LCK, LAT, and ZAP-70 were evaluated in CD4+ TL lysates from pediatric healthy donors, HAV-seropositive pediatric patients with CB levels between 0.3 and 2 mg/dL, and HAV-seropositive pediatric patients with CB > 2 mg/dL under basal conditions (Figure 2(a)). No changes in the degree of phosphorylation for ERK, LCK, LAT, and ZAP-70 were found. In contrast, a significant increase in the MFI of phosphorylated CREB in patients with CB between 0.3 and 2 mg/dL (77.40 ± 60.44) compared with patients with CB > 2 mg/dL (28.79 ± 3.414) and healthy controls (29.75 ± 4.665) was observed, Figure 2(d). Interestingly, a trend toward a reduction in the MFI of phosphorylated CD3-epsilon and SYK and a significant reduction in the MFI of phosphorylated CREB was found in patients with CB levels > 2 mg/dL compared with patients with CB levels between 0.3 and 2 mg/dL (Figures 2(b)–2(d)). These results suggest that, in the context of HAV infection, an augment in phosphorylation of the TCR signaling pathway occurs and it is not affected by discrete CB levels in the microenvironment, whereas CB levels greater than 2 mg/dL result in a reduced phosphorylation of the TCR signaling pathway.

### 3.3. The Percentage of CD4+CD25+ T Cells in HAV+ Patients Increased with Low Serum CB Concentration

**Figure 3:**
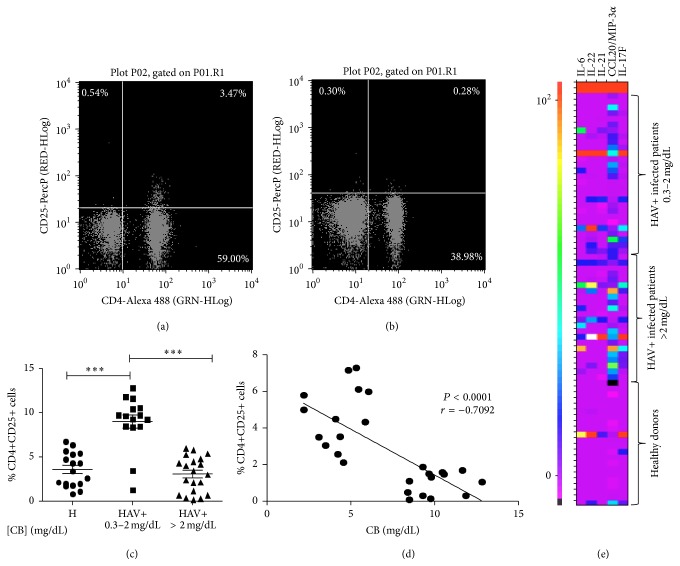
HAV-seropositive patients with low CB levels show an increase in the percentage of CD4+CD25+ TLs. PBLCs were separated from patients and controls. Cells were stained with anti-CD4-Alexa 488 and anti-CD25-PercP and then analyzed by using flow cytometry. Representative dot plot from (a) HAV+ patients with CB 0.3–2 mg/dL and (b) HAV+ patients with CB greater than 2 mg/dL are shown. (c) The results are displayed as the percentage of double positive cells for CD4 and CD25. The medians and the SD from 17 healthy donors, 17 patients with CB 0.3–2 mg/dL, and 15 patients with CB greater than 2 mg/dL are presented. Nonparametric Kruskal-Wallis for comparison between groups was used to calculate statistical significance. *P* < 0.05 was considered statistically significant. ^*∗∗∗*^ *P* < 0.0001. The Spearman correlation coefficient for the percentage of CD4+CD25+ T cells and the CB levels in HAV+ patients was calculated in (d). (e) Th17 cytokines were evaluated in serum from patients with CB 0.3–2 mg/dL (*n* = 44), patients with CB greater than 2 mg/dL (*n* = 45) and healthy donors (*n* = 40) by MAGPIX Technology. Representative Heat map (pg/mL) of 40 controls and 71 patients is shown.

Previous data show differences relative to the intracellular activity of CD4+ TLs based on the CB concentrations present in the medium. To have an overview of the potential role of CB on Tregs subpopulation in HAV+ patients, anti-CD4 and anti-CD25 staining were performed in pediatric patients and pediatric healthy controls. Patients diagnosed with acute HAV infection and presenting CB values below 2 mg/dL show a significantly increased percentage of CD4+CD25+ T cells (8.995 ± 3.006) compared with patients with CB levels > 2 mg/dL (3.068 ± 1.992) and healthy controls (3.546 ± 1.928) (Figures 3(a), 3(b), and 3(c)). This observation agrees with the analysis of CD4+ cells from HAV+ patients with distinct CB levels and stained with anti-CD25 and anti-FOXP3, where we found an increase in the percentage of CD4+CD25+FOXP3+ cells in those HAV+ patients with serum CB levels < 2 mg/dL (data not shown). Moreover, a trend toward a negative correlation between the percentage of CD4+CD25+ T cells and CB levels in HAV+ patients was found (Figure 3(d)). A Th17 profile characterized for increased levels of IL-6, IL-21, IL-22, CCL20, and IL-17F was found in patients with CB > 2 mg/dL (Figure 3(e)). Altogether, these data suggest that the proportion of Tregs is essential in the modulation of the inflammatory process activated during HAV infection and subsequently that Tregs proportion may be related to the serum CB concentration.

### 3.4. Tregs Activity Is Augmented after CB Treatment* In Vitro*

**Figure 4:**
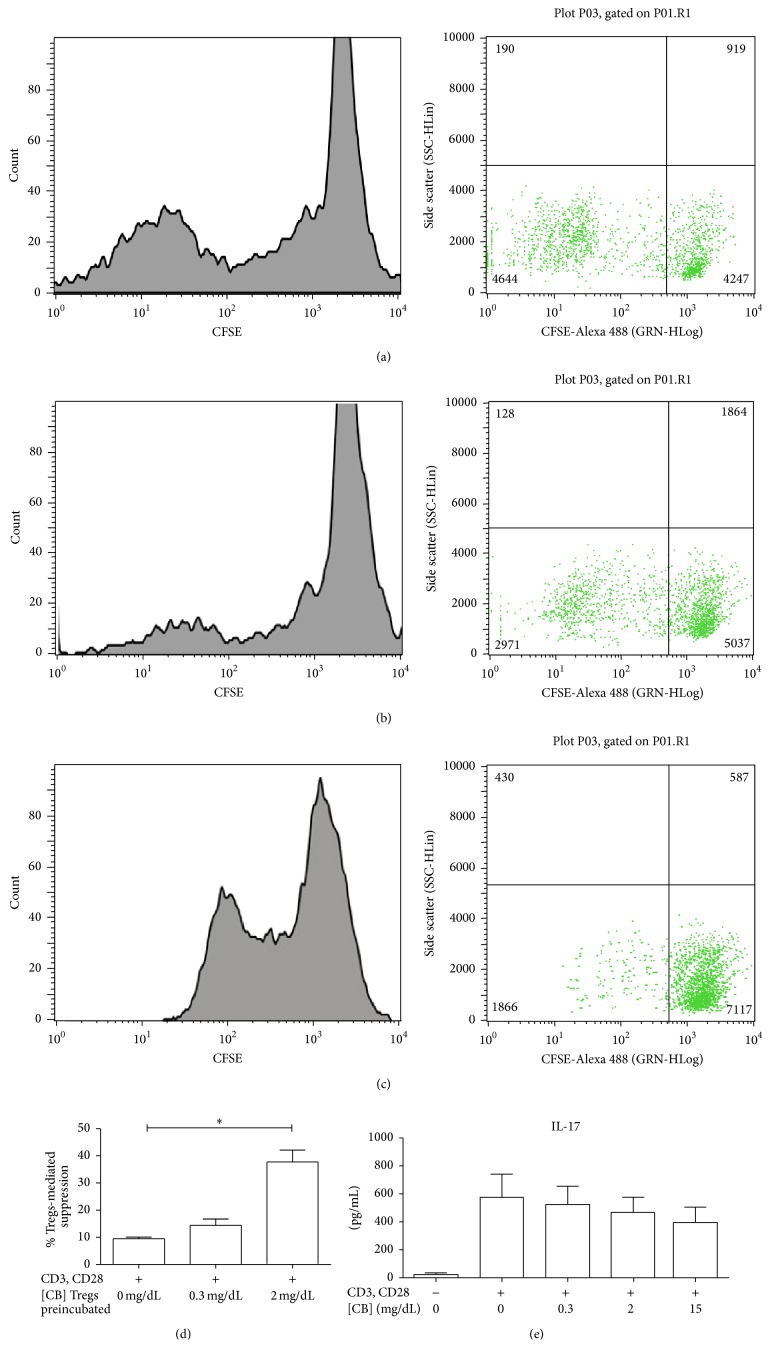
Treatment with CB* in vitro* induces an augment in the activity of Tregs. Purified CD4+CD25+FOXP3+ Tregs from pediatric healthy donors were incubated for 72 hours with and without different doses of CB. Then, Tregs were cocultured with CFSE-labeled Teff (TCD4+CD25−) for 7 days, and cells were stimulated with anti-CD3 and anti-CD28. (a) Dilution of fluorescence and cell count because of the proliferation of Teff stimulated with anti-CD3 and anti-CD28 cocultured with Tregs in the absence of CB. (b) Dilution fluorescence and cell count because of the proliferation of Teff when cocultured with Tregs with CB 0.3 mg/dL. (c) Dilution fluorescence and cell count because of the proliferation of Teff when cocultured with Tregs preincubated with CB 2 mg/dL. Data shown are representative of three independent experiments. (d) The results are displayed as the percentage of Tregs suppression by each condition. (e) IL-17 concentration was evaluated by MAGPIX Technology in cell supernatants from CD4+ TLs from pediatric healthy donors stimulated with anti-CD3 and anti-CD28 in presence or absence of CB. The medians and standard deviations of three repetitions are presented. Nonparametric Kruskal-Wallis for comparison between groups was used to calculate statistical significance. *P* < 0.05 was considered statically significant. ^*∗*^ *P* < 0.05.

To determine the potential role of CB in modulating the activity of Treg cells, coculture assays were performed with Tregs from pediatric healthy donors that were pretreated with and without CB and their autologous CFSE-labeled Teffs in the presence of anti-CD3 and anti-CD28 mAbs. Efficient Teff proliferation (nonsuppressive activity) was observed after stimulation with anti-CD3 and anti-CD28 in the absence of Tregs (data not shown). In contrast, a trend toward increased suppressive activity of Tregs was observed when pretreated with 0.3 mg/dL CB relative to Tregs without CB pretreatment (Figures 4(a), 4(b) and 4(d)). A significant increase in Treg suppressive activity was observed after treatment with 2 mg/dL CB relative to Tregs without CB pretreatment (Figures 4(c) and 4(d)). Moreover, in patients with low CB levels (0.3–2 mg/dL), Tregs appear to be less active, supporting that CB values greater than 2 mg/dL result in increased Treg activity. No differences in IL-17 levels were found in supernatants from TLs treated with anti-CD3, anti-CD28, and different doses of CB relative to TLs treated with anti-CD3 and anti-CD28 without CB (Figure 4(e)), suggesting that CB does not induce a Th17 profile. Thus, our data support that the effect of CB is on Tregs function and this may be related to the efficient control of the inflammatory process activated during HAV infection.

### 3.5. Following CB Treatment* In Vitro*, TIM-1 Expression in Tregs Is Augmented

**Figure 5:**
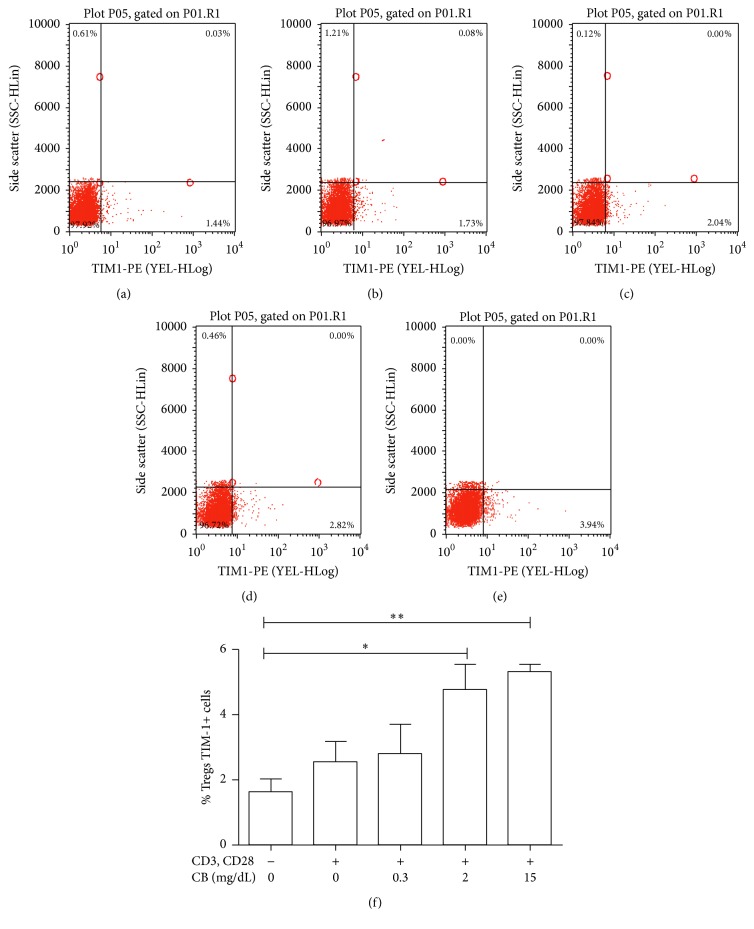
CB* in vitro* induces an augment in the percentage of Tregs expressing TIM-1. Purified Tregs from pediatric healthy donors were incubated at 37°C, 5% CO_2_ with different concentrations of CB for 72 h. Then, cells were recovered and stained with anti-TIM-1-PE and the percentage of Tregs expressing TIM-1 was analyzed by using flow cytometry. Representative dot plots are shown. (a) Tregs without stimulus. (b) Tregs stimulated with anti-CD3 and anti-CD28. (c) Tregs stimulated with anti-CD3, anti-CD28 and CB (0.3 mg/dL). (d) Tregs stimulated with anti-CD3, anti-CD28, and CB (2 mg/dL). (e) Tregs stimulated with anti-CD3, anti-CD28, and CB (15 mg/dL). (f) The results are presented as TIM-1 percentage. The medians and SD of three independent experiments are shown. Nonparametric Kruskal-Wallis for comparison between groups was used to calculate statistical significance. ^*∗*^ *P* < 0.05 was considered statistically significant. ^*∗∗*^ *P* < 0.001.

As our data pointed out that CB upregulates the numbers and the activity of Treg cells we investigated the possible mechanism responsible for this; particularly, we assessed whether changes in TIM-1 expression on the Tregs surface may influence the interaction between the virus and cell and the consequent functionality/activity of these cells during a specific period of the infection. Herein, the effect of CB on the relative proportion of TIM-1 on Tregs was evaluated. Tregs purified from pediatric healthy donors were treated with varying concentrations of CB and stimulated with anti-CD3 and anti-CD28 mAbs. Then, cells were stained to determine possible changes in the expression of TIM-1 because of CB addition. A significant increase in the percentage of TIM-1 expression in Tregs treated with CB (2 mg/dL) (4.783 ± 1.341) and CB (15 mg/dL) (5.333 ± 0.3803) (Figure 5(f)) compared with control (unstimulated cells without CB) (1.620 ± 0.7238) (Figure 5(a)) was found. Additionally, the percentage of cells expressing TIM-1 increased as the concentration of CB increased (Figures 5(c)–5(f)). These data suggest that CB levels have an effect on the expression of TIM-1 in Tregs.

### 3.6. HAV Infection Leads to an Increase in the Number of CD4+CD25+ T Cells Expressing TIM-1

**Figure 6:**
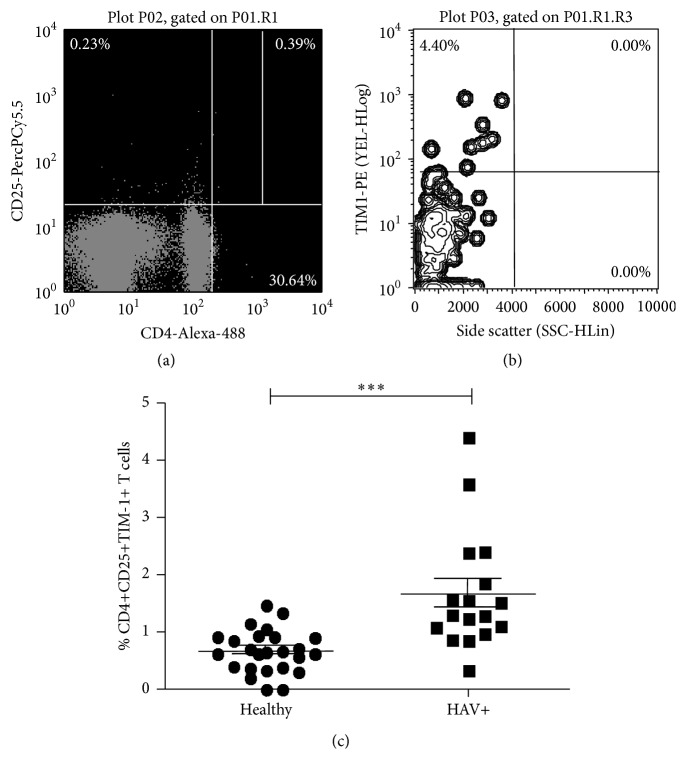
HAV infection leads to an increase in the relative proportion of TIM-1 receptor on CD4+CD25+ TLs. PBLCs from HAV+ pediatric patients were stained with anti-CD4-Alexa 488, anti-CD25-PerCP and anti-TIM1-PE antibodies and evaluated by using flow cytometry. (a) Representative dot plot of CD4+CD25+ staining and selection of the right upper quadrant. (b) Representative dot plot of TIM-1 versus Side Scatter linked to the right upper quadrant on (a), which represents TIM-1 percentage on CD4+CD25+ cells. (c) The results are displayed as the percentage of TIM-1+CD4+CD25+ T cells. The medians and standard deviations from 25 healthy donors and 17 HAV-infected pediatric patients are presented. Nonparametric Mann-Whitney *U* test for comparison between groups was used to calculate statistical significance. *P* < 0.05 was considered statically significant. ^*∗∗∗*^ *P* < 0.0001.

To determine whether the proportion of CD4+CD25+ T cells expressing TIM-1 changes during the course of HAV infection, samples from healthy donors and HAV+ patients were analyzed* ex vivo* as outlined in Figure 6. The percentage of CD4+CD25+ TIM-1 positive cells was significantly higher in HAV-infected pediatric patients (1.658 ± 0.25) compared with healthy controls (0.6628 ± 0.07482) Figure 6(c). Given the acute status of the infection in these patients (all of them had CB > 0.3 mg/dL), these data are consistent with the* in vitro* results: CB modulates the proportion of CD4+CD25+TIM-1+ T cells, even in the presence of the virus.

### 3.7. In Healthy Donors and HAV-Seropositive Patients the 157insMTTTVP Insertion in the HAVCR1/TIM-1 Gene Is Found at a Low Frequency

The 157insMTTTVP polymorphism located in exon 4 of the gene* HAVCR1/TIM-1* has recently been associated with the development of fulminant hepatitis A in an Argentinean population [23]. To determine if the presence of this polymorphism and the activity of Treg cells during HAV infection could be related, the frequency of this polymorphism in a control group and a group of HAV+ pediatric patients was estimated. Gene sequencing of exon 4 was performed and aligned against reference sequences from GenBank in a cohort, which included a total of 99 control samples (98 sequences obtained with primer Forward, 93 sequences obtained with primer Reverse) and 21 HAV-seropositive patients (20 sequences Forward, 21 Reverse sequences).

Two insertional events in the same position, one of 15 nucleotides (ATG ACG ACT GTT CCA) and one of 18 nucleotides (ATG ACA ACG ACT GTT CCA), encoding the amino acids sequences MTTVP and MTTTVP, respectively, were found. In the case of the controls, two samples had the 18-nucleotide insertion, both confirmed for each DNA strand. In the case of HAV-seropositive patients, one sample was found with the 18-nucleotide insertion. The proportions of samples with the insertion are shown in Table 2. Overall, a frequency of 8.08% for the control group and of 9.56% for HAV-seropositive patients was found (Table 2). The odds ratio was 1.19, (with a confidence interval of 0.2340–6.0515). This low frequency of 157insMTTTVP insertion in the* HAVCR1/TIM-1* gene in healthy donors and HAV-seropositive patients suggests that no association is observed between this polymorphism in the viral receptor and symptoms of HAV-related disease. Thus, this gene polymorphism is present in Mexican population but does not seem to be playing a role in the susceptibility to HAV infection.

## 4. Discussion

The results obtained in this study support the hypothesis that CB plays an important role in modulating the functionality of immune system cells during the infectious process mediated by HAV. The resolution of the infection caused by HAV is influenced by the dynamics of T lymphocytes, particularly CD4+ TLs and Tregs [27]. During HAV infection, CD4+ TLs participate in cytokine secretion and helper functions to eradicate the virus [28]; these functions are directly related to the activation of the TCR signaling pathway [25]. Signals transduced by CD3 epsilon contribute to the survival of T cells [26]. In these cells, CREB activation promotes proliferation, survival, and differentiation by regulating the Th1, Th2, and Th17 responses, in addition to the signaling cascades required for the generation and maintenance of Tregs [29]. Moreover, SYK efficiently phosphorylate components of the TCR signaling cascade, acting as a positive regulator [30]. Our results support the idea that CB acts directly on this pathway, modulating the functionality of CD4+ TLs. Different responses were noticed when* in vitro* and* ex vivo* data were compared regarding the dose-response effect of CB on intracellular signals. This may be because overall phosphorylation was detected with an anti-pTyr antibody for the analysis* in vitro* whereas specific signaling components were evaluated* ex vivo*. The* in vitro* analysis of CD4+ TLs showed a lower degree of tyrosine phosphorylation in cells treated with CB (between 0.3 mg/dL to 15 mg/dL) than in controls (Figure 1). This is in agreement with previous studies [31], which indicate that BR inhibits the activity of the catalytic domain of protein kinases via a noncompetitive mechanism. In contrast, in patients with low levels of CB the degree of phosphorylation was not affected in our study, as patients showed a rise in the degree of phosphorylation of CREB. This suggests that, in the context of HAV infection, an augment in phosphorylation of the TCR signaling pathway occurs and discrete CB levels in the microenvironment do not affect it. Interestingly, our* ex vivo* analysis showed that patients with CB > 2 mg/dL had a lower degree of phosphorylation of proteins involved in the TCR pathway such as CD3 epsilon, SYK, and CREB (Figure 2). This suggests that the activity of these particular proteins is diminished during the course of HAV infection and CB levels may be related to this process.

To have an overview of the potential role of CB on Tregs subpopulation, we investigated the percentage of CD4+CD25+ TLs in HAV-seropositive patients with various serum CB levels. The results indicated an inverse relationship between the level of CB and the percentage of CD4+CD25+ T cells (Figure 3). This could be explained by the mechanism proposed by Sakaguchi et al. [32], who identified a specific demethylated region in the FOXP3 locus of Tregs, which contains a binding site for the CREB transcription factor, indicating that CREB stabilizes FOXP3 expression and thus promotes and maintains the Treg populations. In our study, increased CREB phosphorylation was observed only in CD4+ TLs from patients with low CB levels. This finding suggests that the concentration of this bile metabolite in the medium is crucial for the development of CD4+ TLs and potentially regulates Treg population size and/or function. In addition, several authors have reported the relationship of BR and Tregs, but the reports are contradictory. First, Liu et al., 2008, reported that* in vitro* BR treatment of CD4+ T cells did not induce an expansion of Tregs [3]. In contrast, Rocuts et al., 2010, established that treatment with BR administered to murine allograft pancreatic islets recipients promotes* de novo* generation of Tregs [6], which leads to tolerization after the administration of BR in transplant recipients [33]. Moreover, Huang et al., 2015, reported that an increase in the proportion of Tregs with memory phenotype and TNFRII high expression in cirrhotic patients correlated with hyperbilirubinemia [34]. The discrepancy in results is most likely due to the differences in methodologies and models used in each of the studies. Although our data support the notion that CB may promote* de novo* generation of Tregs by modulating intracellular signals, specifically CREB phosphorylation, preliminary data from* in vitro* CB treatment of naïve TLs revealed no differences in FOXP3 expression (data not shown), suggesting that, under these particular experimental conditions, CB does not promote* de novo* generation of Tregs. Large-scale studies are necessary to dissect the functional significance of various concentrations of CB in Tregs differentiation in the context of HAV infection. Particularly, a detailed study of the intracellular signals modulated in Tregs because of CB treatment deserves detailed analysis. Altogether, our* in vitro* data strongly suggest that CB > 2 mg/dL increases the suppressive capacity of Tregs (Figure 4), thus contributing to the nonoptimal functional status of CD4+ TLs present during the HAV infection. This is in agreement with the anti-inflammatory properties of BR previously described and with the fact that, under our experimental conditions, CB did not induce IL-17 secretion in TLs (Figure 4). Of interest is the fact that Th17 differentiation requires TCR activation signaling pathway. In our study, a Th17 profile is found in HAV-infected patients with CB greater than 2 mg/dL (Figure 3). Thus, it is plausible that Th17 profile results of ERK, LAT, LCK, and ZAP-70 signals activated in T cells in the presence of the virus, given no differences relative to CB levels, were found in these particular pathways (Figure 2(a)). In addition, Th17 differentiation requires IL-6 and we recently reported an augment in this cytokine in HAV-infected patients with greater CB levels [17]. Given Th17 profile was assessed in serum from patients, this particular profile could result from IL-6 secretion from macrophages.

There is evidence that the antiproliferative activity of Tregs is regulated during the HAV infection. This has been mainly attributed to the interaction generated between the virus and the HAVCR1/TIM-1 receptor expressed on Tregs [20]. Changes in the proportion of TIM-1 may modulate the degree of the activation of this cell subtype. Discrete concentrations of BR suppress the reactivity of CD4+ T cells through mechanisms, including inhibition of CD28, B7-1, and B7-2, resulting in a reduction of costimulatory signals [3]. Interestingly, intraperitoneal administration of BR in mice influences the expression of Fc receptors in macrophages [35]. These data support the hypothesis that BR could modulate immune functions, due to its lipophilic character and direct interaction with cell membranes, suggesting that BR is associated with membrane receptors. The results of these studies coincide with the results obtained in the present study, which indicate that CB is able to modulate the proportion of Tregs expressing the HAVCR1/TIM-1 receptor* in vitro* (Figure 5), as well as the data obtained by the* ex vivo* analysis in the presence of HAV infection (Figure 6). Higher levels of CB result in a greater proportion of HAVCR1/TIM1-positive Tregs. This coincides with increased suppressive function of these Tregs, and thus, it is plausible that CB modulates the function of Tregs via the expression of HAVCR1/TIM-1. Moreover, based on a small population, the 157insMTTTVP insertion in the* HAVCR1/TIM-1* gene found in low frequency did not support a functional association between genetic differences at the host level and the presence of HAV infection. A larger population is required in order to better assess the contribution of gene polymorphisms in the viral receptor to the development of clinical outcomes during the HAV infectious process.

In conclusion, our data strongly suggest that, during HAV infection, CB plays a role in determining T cell function by modulating intracellular pathways and by inducing changes in the function of Tregs in mechanisms related to the expression of HAVCR1/TIM-1 on the cell surface. The CB levels found to play a role in viral hepatitis infection might provide insights for other infectious diseases eventually affected by host metabolites at the level of regulating immune responses. HAV and its interaction with the immune system represent a field for future investigation. In particular, exploring the nature of these interactions may contribute to understand why this virus does not persist in the infected host, whereas other viruses, including hepatitis B and hepatitis C, do.

## Figures and Tables

**Table 1:** Demographic and clinical characteristics of HAV-infected pediatric patients and controls.

Characteristic	Healthy controls (*n* = 60)	Patients		*P* value
		HAV+ CB: >0.3–2 mg/dL (*n* = 70)	HAV+ CB: >2 mg/dL (*n* = 86)	
Gender (% female)	41	58	56	NS
Mean age (years ± SD)	6.5 ± 2.5	6.5 ± 3.425	8.05 ± 3.85	NS
Mean ALT (IU/L ± SD)	21.58 ± 13.72	733.39 ± 629.50	1471.06 ± 1238.43	NS
Mean AST (IU/L ± SD)	13.87 ± 9.99	513.94 ± 493.8	1041.06 ± 935.33	<0.05
Mean CB (mg/dL ± SD)	0.20 ± 0.087	1.15 ± 0.70	5.33 ± 2.65	—
Anti-HAV IgM	−	+	+	—
Anti-HAV IgG	−	−	−	—

ALT: alanine aminotransferase; AST: aspartate aminotransferase; SD: standard deviation; NS: not significant.

**Table 2:** The frequency of the 157insMTTTVP insertion in *HAVCR1/TIM1* gene in healthy donors and HAV-infected patients.

	Control group	HAV-infected pediatric patients
Total samples	99	21
Samples with insertion (2 alleles)	2	1
Samples with insertion (1 allele)	6	1
Percentage	8.06	9.52
